# Bis{2-[(4-chloro­phen­yl)imino­meth­yl]pyrrol-1-ido-κ^2^ *N*,*N*′}bis­(dimethyl­amido-κ*N*)titanium(IV) toluene monosolvate

**DOI:** 10.1107/S1600536812014961

**Published:** 2012-04-18

**Authors:** Zhou Chen, Jian Wu, Yahong Li, Bin Hu

**Affiliations:** a Qinghai Institute of Salt Lakes, Chinese Academy of Sciences, Xining 810008, People’s Republic of China; b Key Laboratory of Organic Synthesis of Jiangsu Province, College of Chemistry, Chemical Engineering and Materials Science, Soochow University, Suzhou 215123, People’s Republic of China

## Abstract

The mononuclear title compound, [Ti(C_11_H_8_ClN_2_)_2_(C_2_H_6_N)_2_]·C_7_H_8_, was synthesized by the reaction of *N*-(4-chloro­phen­yl)-2-pyrrolylcarbaldimine with Ti(C_2_H_6_N)_4_. The Ti^IV^ ion is situated on a twofold rotation axis and displays a distorted octa­hedral geometry defined by four N atoms from two 2-[(4-chloro­phen­yl)imino­meth­yl]pyrrol-1-ide ligands and two N atoms from two dimethyl­amine ligands. The Ti—N_pyrrole_ bond length [2.1041 (19) Å] is longer than the Ti—N_dimethyl­amine_ bond length [1.9013 (19) Å]; the imine N atom exhibits the longest Ti—N bond [2.3152 (17) Å]. The toluene solvent mol­ecule is located on a twofold rotation axis running through the C atom of the methyl group. Consequently, the H atoms of the latter are rotationally disordered. The compound contains no markable hydrogen-bonding inter­actions.

## Related literature

For the synthesis of *N*-(4-chloro­phen­yl)-2-pyrrolylcarbald­imine and its oxidovanadium(IV) complexes, see: Mozaffar *et al.* (2010 ▶). For the synthesis of titanium amido complexes and their applications in hydro­amination reactions, see: Ramanathan *et al.* (2004 ▶); Cao *et al.* (2001 ▶); Bexrud *et al.* (2007 ▶); Tillack *et al.* (2005 ▶); Braunschweig & Breitling (2006 ▶); Zhao *et al.* (2012 ▶).
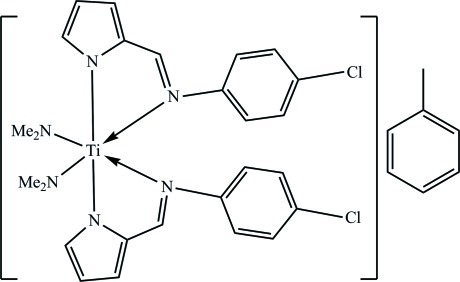


## Experimental

### 

#### Crystal data

[Ti(C_11_H_8_ClN_2_)_2_(C_2_H_6_N)_2_]·C_7_H_8_
*M* *_r_* = 635.48
Orthorhombic, 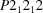

*a* = 11.1952 (4) Å
*b* = 13.8545 (6) Å
*c* = 10.4651 (3) Å
*V* = 1623.18 (10) Å^3^
*Z* = 2
Mo *K*α radiation
μ = 0.46 mm^−1^
*T* = 296 K
0.27 × 0.25 × 0.20 mm

#### Data collection

Bruker APEXII CCD diffractometer
Absorption correction: multi-scan (*SADABS*; Bruker, 2005 ▶) *T*_min_ = 0.886, *T*_max_ = 0.914
7377 measured reflections
3172 independent reflections
2855 reflections with *I* > 2σ(*I*)
*R*_int_ = 0.021

#### Refinement

*R*[*F* ^2^ > 2σ(*F* ^2^)] = 0.035
*wR*(*F* ^2^) = 0.103
*S* = 1.04
3172 reflections
191 parameters
H-atom parameters constrained
Δρ_max_ = 0.24 e Å^−3^
Δρ_min_ = −0.81 e Å^−3^
Absolute structure: Flack (1983 ▶), 1338 Friedel pairs
Flack parameter: 0.00 (3)

### 

Data collection: *APEX2* (Bruker, 2005 ▶); cell refinement: *SAINT* (Bruker, 2005 ▶); data reduction: *SAINT*; program(s) used to solve structure: *SHELXS97* (Sheldrick, 2008 ▶); program(s) used to refine structure: *SHELXL97* (Sheldrick, 2008 ▶); molecular graphics: *SHELXTL* (Sheldrick, 2008 ▶); software used to prepare material for publication: *SHELXTL*.

## Supplementary Material

Crystal structure: contains datablock(s) I, global. DOI: 10.1107/S1600536812014961/wm2614sup1.cif

Structure factors: contains datablock(s) I. DOI: 10.1107/S1600536812014961/wm2614Isup2.hkl

Additional supplementary materials:  crystallographic information; 3D view; checkCIF report

## Figures and Tables

**Fig. 1.**
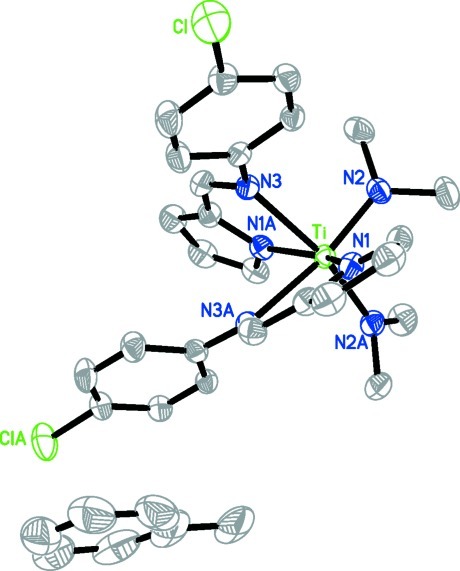
The molecular structure of (I), with atom labels and displacement ellipsoids at the 30% probability level. [Symmetry code: A) -x + 1, -y, z.]

**Fig. 2.**
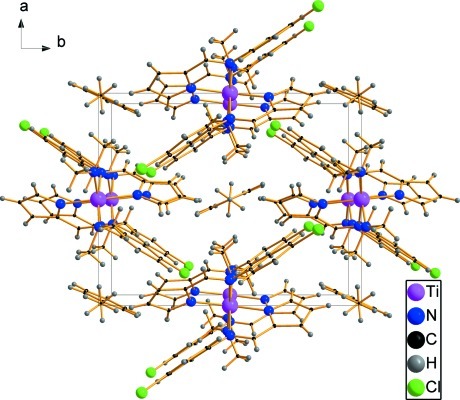
The packing diagram of the compound in a view down [001].

## supplementary crystallographic information

### Comment

The ligand *N*-(4-chlorophenyl)-2-pyrrolylcarbaldimine can be synthesized by the reaction of 4-chloroaniline and 2-pyrrolaldehyde. The ligand has been used in the synthesis of oxidovanadium(IV) complexes (Mozaffar *et al.*, 2010). Herein we report the synthesis and crystal structure of a titanium amido complex [Ti(C_11_H_8_N_2_Cl)_2_(C_2_H_6_N)_2_](C_6_H_5_CH_3_), (I). Such titanium amido complexes were employed as catalysts in the hydroamination of alkynes (Ramanathan *et al.*, 2004; Cao *et al.*, 2001; Bexrud *et al.*, 2007; Tillack *et al.*, 2005; Braunschweig & Breitling, 2006; Zhao *et al.*, 2012).

The molecular structure of (I) is shown in Fig. 1. The Ti^IV^ ion has site symmetry 2 and displays a distorted octahedral geometry. It is coordinated by four N atoms from two symmetry-related bidentate *N*-(4-chlorophenyl)-2-pyrrolylcarbaldimine ligands and two nitrogen atoms from two dimethylamino ions. Two pyrrolyl N atoms from two coordinating *N*-(4-chlorophenyl)-2-pyrrolylcarbaldimine molecules occupying *trans* positions in the equatorial plane. The dihedral angle between the pyrrolcarbaldiimine and chlorophenyl moieties in the bidentate ligand is 44.90 (10)° . There is a solvate toluene molecule present that is also located on a twofold rotation axis. Since the methyl group of the solvate toluene lies on a special position of higher symmetry than the molecular can possess, the H atoms of this group are rotationally disordered.

The compound contains no remarkable hydrogen bonding interactions. In the crystal packing, the complexes form channels parallel to [001] where the solvent molecules are located.

### Experimental

To a solution of Ti(NMe_2_)_4_ (0.112 g, 0.5 mmol) in THF (2 ml) was added *N*-(4-chlorophenyl)-2-pyrrolylcarbaldimine (0.204 g, 1 mmol) in THF (3 ml). After stirring at room temperature overnight, volatiles were removed in vacuo, resulting in an orange solid (0.246 g, 91%). Single crystals of (I) were grown from a toluene/hexane (1:1) solution at 238 K.

### Refinement

All H atoms were placed in geometrically idealized positions and constrained to ride on their parent atoms with C—H = 0.93 Å for aromatic H atoms, 0.96 Å for CH_3_ type H atoms and 0.98 Å for CH type H atoms, respectively. *U*_iso_(H) values were set at 1.5Ueq(C) for methyl H atoms, and 1.2Ueq(C) for the rest of the H atoms. The methyl group of the solvent molecule lies on a twofold rotation axis; consequently, the H atoms of this methyl group are disordered and were refined with an occupancy of 0.5.

### Crystal data

**Table:**

[Ti(C_11_H_8_ClN_2_)_2_(C_2_H_6_N)_2_]·C_7_H_8_	*F*(000) = 664
*M**_r_* = 635.48	*D*_x_ = 1.300 Mg m^−^^3^
Orthorhombic, *P*2_1_2_1_2	Mo *K*α radiation, λ = 0.71073 Å
Hall symbol: P 2 2ab	Cell parameters from 3510 reflections
*a* = 11.1952 (4) Å	θ = 2.7–25.3°
*b* = 13.8545 (6) Å	µ = 0.46 mm^−^^1^
*c* = 10.4651 (3) Å	*T* = 296 K
*V* = 1623.18 (10) Å^3^	Block, red
*Z* = 2	0.27 × 0.25 × 0.20 mm

### Data collection

**Table:**

Bruker APEXII CCD diffractometer	3172 independent reflections
Radiation source: fine-focus sealed tube	2855 reflections with *I* > 2σ(*I*)
Graphite monochromator	*R*_int_ = 0.021
φ and ω scans	θ_max_ = 26.0°, θ_min_ = 2.0°
Absorption correction: multi-scan (*SADABS*; Bruker, 2005)	*h* = −13→13
*T*_min_ = 0.886, *T*_max_ = 0.914	*k* = −6→17
7377 measured reflections	*l* = −12→12

### Refinement

**Table:**

Refinement on *F*^2^	Secondary atom site location: difference Fourier map
Least-squares matrix: full	Hydrogen site location: inferred from neighbouring sites
*R*[*F*^2^ > 2σ(*F*^2^)] = 0.035	H-atom parameters constrained
*wR*(*F*^2^) = 0.103	*w* = 1/[σ^2^(*F*_o_^2^) + (0.0643*P*)^2^ + 0.167*P*] where *P* = (*F*_o_^2^ + 2*F*_c_^2^)/3
*S* = 1.04	(Δ/σ)_max_ < 0.001
3172 reflections	Δρ_max_ = 0.24 e Å^−^^3^
191 parameters	Δρ_min_ = −0.81 e Å^−^^3^
0 restraints	Absolute structure: Flack (1983), 1338 Friedel pairs
Primary atom site location: structure-invariant direct methods	Flack parameter: 0.00 (3)

### Special details

**Table:**

Geometry. All e.s.d.'s (except the e.s.d. in the dihedral angle between two l.s. planes) are estimated using the full covariance matrix. The cell e.s.d.'s are taken into account individually in the estimation of e.s.d.'s in distances, angles and torsion angles; correlations between e.s.d.'s in cell parameters are only used when they are defined by crystal symmetry. An approximate (isotropic) treatment of cell e.s.d.'s is used for estimating e.s.d.'s involving l.s. planes.
Refinement. Refinement of *F*^2^ against ALL reflections. The weighted *R*-factor *wR* and goodness of fit *S* are based on *F*^2^, conventional *R*-factors *R* are based on *F*, with *F* set to zero for negative *F*^2^. The threshold expression of *F*^2^ > σ(*F*^2^) is used only for calculating *R*-factors(gt) *etc*. and is not relevant to the choice of reflections for refinement. *R*-factors based on *F*^2^ are statistically about twice as large as those based on *F*, and *R*- factors based on ALL data will be even larger.

### Fractional atomic coordinates and isotropic or equivalent isotropic displacement parameters (Å^2^)

**Table:**

	*x*	*y*	*z*	*U*_iso_*/*U*_eq_	Occ. (<1)
Ti	0.5000	0.0000	−0.00125 (4)	0.03946 (15)	
N3	0.37242 (16)	−0.00104 (15)	0.17286 (15)	0.0444 (4)	
N1	0.52139 (16)	0.14665 (13)	0.04575 (18)	0.0468 (4)	
C7	0.32052 (19)	0.07660 (18)	0.2402 (2)	0.0438 (5)	
N2	0.37235 (18)	0.00986 (16)	−0.12037 (17)	0.0511 (4)	
C8	0.25902 (18)	0.14699 (16)	0.1737 (2)	0.0458 (5)	
H8	0.2554	0.1440	0.0850	0.055*	
C9	0.2030 (2)	0.22161 (18)	0.2368 (2)	0.0515 (5)	
H9	0.1606	0.2681	0.1915	0.062*	
C2	0.5137 (3)	0.30613 (18)	0.0851 (3)	0.0651 (7)	
H2	0.4953	0.3712	0.0762	0.078*	
C13	0.3605 (2)	−0.08821 (17)	0.2195 (2)	0.0506 (5)	
H13	0.3171	−0.0985	0.2940	0.061*	
C1	0.4781 (2)	0.23225 (17)	0.0050 (2)	0.0551 (6)	
H1	0.4307	0.2404	−0.0672	0.066*	
C12	0.3297 (2)	0.0845 (2)	0.3729 (2)	0.0594 (7)	
H12	0.3734	0.0392	0.4187	0.071*	
C11	0.2741 (3)	0.1593 (2)	0.4364 (2)	0.0673 (7)	
H11	0.2796	0.1642	0.5248	0.081*	
C3	0.5818 (3)	0.2653 (2)	0.1808 (3)	0.0642 (7)	
H3	0.6179	0.2972	0.2487	0.077*	
C10	0.2107 (2)	0.22629 (19)	0.3680 (2)	0.0549 (6)	
C5	0.2535 (2)	−0.0300 (2)	−0.1037 (3)	0.0651 (7)	
H5A	0.1973	0.0215	−0.0914	0.098*	
H5B	0.2526	−0.0716	−0.0304	0.098*	
H5C	0.2318	−0.0663	−0.1784	0.098*	
C4	0.5856 (2)	0.16622 (17)	0.1551 (2)	0.0505 (5)	
C14	1.0000	0.0000	0.3423 (7)	0.135 (2)	
C15	0.9586 (4)	0.0770 (4)	0.4130 (6)	0.1245 (18)	
H15	0.9309	0.1311	0.3696	0.149*	
C16	0.9561 (4)	0.0779 (4)	0.5446 (6)	0.1265 (17)	
H16	0.9251	0.1304	0.5888	0.152*	
C6	0.3784 (3)	0.0670 (2)	−0.2366 (3)	0.0746 (8)	
H6A	0.3631	0.0264	−0.3091	0.112*	
H6B	0.4565	0.0950	−0.2444	0.112*	
H6C	0.3196	0.1174	−0.2332	0.112*	
C17	1.0000	0.0000	0.6079 (7)	0.117 (2)	
H17	1.0000	0.0000	0.6968	0.141*	
Cl	0.13535 (9)	0.31837 (6)	0.44888 (7)	0.0879 (3)	
C18	1.0000	0.0000	0.2026 (6)	0.135 (2)	
H18A	0.9518	0.0524	0.1720	0.203*	0.50
H18B	0.9679	−0.0600	0.1720	0.203*	0.50
H18C	1.0803	0.0075	0.1720	0.203*	0.50

### Atomic displacement parameters (Å^2^)

**Table:**

	*U*^11^	*U*^22^	*U*^33^	*U*^12^	*U*^13^	*U*^23^
Ti	0.0425 (3)	0.0420 (3)	0.0339 (2)	0.0016 (2)	0.000	0.000
N3	0.0455 (9)	0.0466 (9)	0.0413 (8)	−0.0013 (10)	0.0007 (7)	0.0020 (9)
N1	0.0506 (10)	0.0436 (10)	0.0464 (9)	−0.0002 (8)	0.0061 (8)	0.0025 (8)
C7	0.0420 (10)	0.0498 (12)	0.0397 (10)	−0.0017 (10)	0.0044 (9)	0.0029 (9)
N2	0.0529 (10)	0.0574 (12)	0.0431 (8)	0.0072 (10)	−0.0077 (8)	0.0005 (9)
C8	0.0482 (12)	0.0519 (13)	0.0374 (10)	−0.0034 (10)	0.0014 (9)	0.0001 (9)
C9	0.0559 (13)	0.0489 (13)	0.0497 (13)	0.0044 (11)	−0.0017 (11)	0.0017 (10)
C2	0.0718 (17)	0.0404 (12)	0.0831 (17)	0.0009 (13)	0.0133 (15)	0.0038 (12)
C13	0.0515 (12)	0.0522 (14)	0.0482 (12)	−0.0038 (11)	0.0060 (10)	0.0076 (10)
C1	0.0579 (13)	0.0496 (13)	0.0579 (12)	0.0034 (10)	0.0104 (13)	0.0122 (11)
C12	0.0698 (15)	0.0671 (17)	0.0412 (12)	0.0118 (13)	−0.0048 (11)	0.0051 (12)
C11	0.091 (2)	0.0747 (18)	0.0364 (11)	0.0136 (16)	−0.0008 (12)	−0.0040 (12)
C3	0.0696 (16)	0.0471 (14)	0.0760 (17)	−0.0076 (12)	0.0056 (14)	−0.0090 (13)
C10	0.0634 (14)	0.0534 (14)	0.0480 (13)	0.0037 (12)	0.0050 (11)	−0.0074 (11)
C5	0.0571 (15)	0.0772 (18)	0.0611 (15)	0.0017 (13)	−0.0155 (12)	−0.0110 (12)
C4	0.0526 (12)	0.0466 (13)	0.0524 (12)	−0.0073 (11)	0.0042 (10)	−0.0051 (10)
C14	0.126 (3)	0.178 (5)	0.102 (3)	−0.114 (4)	0.000	0.000
C15	0.095 (3)	0.124 (4)	0.154 (5)	−0.039 (3)	−0.043 (3)	0.027 (3)
C16	0.094 (3)	0.144 (5)	0.141 (4)	−0.012 (3)	−0.015 (3)	−0.020 (4)
C6	0.087 (2)	0.084 (2)	0.0535 (14)	0.0153 (17)	−0.0109 (15)	0.0142 (14)
C17	0.094 (4)	0.153 (6)	0.104 (4)	0.016 (5)	0.000	0.000
Cl	0.1167 (7)	0.0798 (5)	0.0672 (4)	0.0314 (5)	0.0061 (4)	−0.0202 (4)
C18	0.126 (3)	0.178 (5)	0.102 (3)	−0.114 (4)	0.000	0.000

### Geometric parameters (Å, º)

**Table:**

Ti—N2^i^	1.9013 (19)	C12—H12	0.9300
Ti—N2	1.9013 (19)	C11—C10	1.370 (4)
Ti—N1	2.1041 (19)	C11—H11	0.9300
Ti—N1^i^	2.1042 (19)	C3—C4	1.399 (4)
Ti—N3^i^	2.3152 (17)	C3—H3	0.9300
Ti—N3	2.3152 (17)	C10—Cl	1.748 (3)
N3—C13	1.309 (3)	C5—H5A	0.9600
N3—C7	1.411 (3)	C5—H5B	0.9600
N1—C1	1.350 (3)	C5—H5C	0.9600
N1—C4	1.378 (3)	C4—C13^i^	1.409 (3)
C7—C8	1.382 (3)	C14—C15	1.378 (6)
C7—C12	1.397 (3)	C14—C15^ii^	1.378 (6)
N2—C5	1.452 (3)	C14—C18	1.462 (8)
N2—C6	1.453 (3)	C15—C16	1.377 (8)
C8—C9	1.378 (3)	C15—H15	0.9300
C8—H8	0.9300	C16—C17	1.359 (6)
C9—C10	1.376 (3)	C16—H16	0.9300
C9—H9	0.9300	C6—H6A	0.9600
C2—C3	1.379 (4)	C6—H6B	0.9600
C2—C1	1.382 (4)	C6—H6C	0.9600
C2—H2	0.9300	C17—C16^ii^	1.359 (6)
C13—C4^i^	1.409 (3)	C17—H17	0.9300
C13—H13	0.9300	C18—H18A	0.9600
C1—H1	0.9300	C18—H18B	0.9600
C12—C11	1.378 (4)	C18—H18C	0.9600
			
N2^i^—Ti—N2	98.06 (12)	C7—C12—H12	119.8
N2^i^—Ti—N1	97.88 (8)	C10—C11—C12	119.4 (2)
N2—Ti—N1	99.75 (8)	C10—C11—H11	120.3
N2^i^—Ti—N1^i^	99.76 (8)	C12—C11—H11	120.3
N2—Ti—N1^i^	97.88 (8)	C2—C3—C4	106.3 (3)
N1—Ti—N1^i^	152.96 (10)	C2—C3—H3	126.9
N2^i^—Ti—N3^i^	93.02 (7)	C4—C3—H3	126.9
N2—Ti—N3^i^	168.31 (8)	C11—C10—C9	121.4 (2)
N1—Ti—N3^i^	74.92 (8)	C11—C10—Cl	119.41 (19)
N1^i^—Ti—N3^i^	83.81 (7)	C9—C10—Cl	119.1 (2)
N2^i^—Ti—N3	168.31 (8)	N2—C5—H5A	109.5
N2—Ti—N3	93.02 (7)	N2—C5—H5B	109.5
N1—Ti—N3	83.81 (7)	H5A—C5—H5B	109.5
N1^i^—Ti—N3	74.92 (8)	N2—C5—H5C	109.5
N3^i^—Ti—N3	76.19 (8)	H5A—C5—H5C	109.5
C13—N3—C7	118.36 (19)	H5B—C5—H5C	109.5
C13—N3—Ti	111.21 (16)	N1—C4—C3	109.6 (2)
C7—N3—Ti	129.97 (15)	N1—C4—C13^i^	118.0 (2)
C1—N1—C4	106.1 (2)	C3—C4—C13^i^	132.4 (2)
C1—N1—Ti	137.17 (17)	C15—C14—C15^ii^	115.0 (7)
C4—N1—Ti	116.32 (15)	C15—C14—C18	122.5 (3)
C8—C7—C12	118.8 (2)	C15^ii^—C14—C18	122.5 (3)
C8—C7—N3	119.42 (19)	C16—C15—C14	123.4 (6)
C12—C7—N3	121.8 (2)	C16—C15—H15	118.3
C5—N2—C6	110.5 (2)	C14—C15—H15	118.3
C5—N2—Ti	125.68 (17)	C17—C16—C15	118.2 (6)
C6—N2—Ti	123.6 (2)	C17—C16—H16	120.9
C9—C8—C7	121.0 (2)	C15—C16—H16	120.9
C9—C8—H8	119.5	N2—C6—H6A	109.5
C7—C8—H8	119.5	N2—C6—H6B	109.5
C10—C9—C8	119.0 (2)	H6A—C6—H6B	109.5
C10—C9—H9	120.5	N2—C6—H6C	109.5
C8—C9—H9	120.5	H6A—C6—H6C	109.5
C3—C2—C1	107.2 (2)	H6B—C6—H6C	109.5
C3—C2—H2	126.4	C16^ii^—C17—C16	121.6 (7)
C1—C2—H2	126.4	C16^ii^—C17—H17	119.2
N3—C13—C4^i^	119.1 (2)	C16—C17—H17	119.2
N3—C13—H13	120.5	C14—C18—H18A	109.5
C4^i^—C13—H13	120.5	C14—C18—H18B	109.5
N1—C1—C2	110.8 (2)	H18A—C18—H18B	109.5
N1—C1—H1	124.6	C14—C18—H18C	109.5
C2—C1—H1	124.6	H18A—C18—H18C	109.5
C11—C12—C7	120.3 (2)	H18B—C18—H18C	109.5
C11—C12—H12	119.8		

Symmetry codes: (i) −*x*+1, −*y*, *z*; (ii) −*x*+2, −*y*, *z*.
